# Impact of Chitosan Molecular Weight and Attached Non-Interactive Chains on the Formation of α-Lactalbumin Nanogel Particles

**DOI:** 10.3390/gels3020014

**Published:** 2017-04-26

**Authors:** Juan Du, Young-Hee Cho, Ryan Murphy, Owen Griffith Jones

**Affiliations:** Department of Food Science, Purdue University, 745 Agriculture Mall Drive, West Lafayette, IN 47907, USA; jdu4062@yahoo.com (J.D.); murph170@purdue.edu (R.M.)

**Keywords:** chitosan, α-lactalbumin, complex, copolymer, polysaccharide, protein, poly(ethylene glycol), nanogel

## Abstract

Thermal treatment of protein–polysaccharide complexes will form nanogel particles, wherein the polysaccharide controls nanogel formation by limiting protein aggregation. To determine the impact of the chitosan molecular weight and non-interactive chains on the formation of nanogels, mixtures of α-lactalbumin were prepared with selectively-hydrolyzed chitosan containing covalently-attached polyethylene glycol chains (PEG) and heated near the protein’s isoelectric point to induce formation of nanogels. Turbidity of heated mixtures indicated the formation of suspended aggregates, with greater values observed at higher pH, without attached PEG, and among samples with 8.9 kDa chitosan. Mixtures containing 113 kDa chitosan-PEG formed precipitating aggregates above pH 5, coinciding with a low-magnitude colloidal charge and average hydrodynamic radii > 400 nm. All other tested mixtures were stable to precipitation and possessed average hydrodynamic radii ~100 nm, with atomic force microscopy showing homogeneous distributions of spherical nanogel aggregates. Over all of the tested conditions, attached PEG led to no additional significant changes in the size or morphology of nanogels formed from the protein and chitosan. While PEG may have interfered with the interactions between protein and the 113 kDa chitosan, prompting greater aggregation and precipitation, PEG did not indicate any such interference for shorter chitosan chains.

## 1. Introduction

Colloidal structures can be utilized for many applications in food materials, such as controlled delivery of bioactive compounds, modification of textures, and stabilization of interfaces [1]. One option is to create gelled biopolymers that are physically, chemically, or enzymatically treated so that their ultimate size is on the colloidal scale (100–10,000 nm). For example, microparticulated whey protein is a comminuted protein gel with excellent application as a fat replacer [2]. Another route is to create nanogels and microgels formed by controlled aggregation of whey proteins, particularly by using heat treatment in highly controlled solution conditions so that the resulting aggregate particles have dimensions within the range of 100–1000 nm [3,4]. These structures have been proposed as interfacial stabilizers or components of composite gels, where the physical properties of the nanogels can impact the total system [5].

In the past decade, it was found that nanogels could be formed by heat treatment of protein and polysaccharide mixtures. Specifically, experiments showed that interactions between whey protein and charged polysaccharides will limit thermal aggregation of the protein at pH values near the isoelectric pH [6]. Inhibiting thermal aggregation of the protein by electrostatic interactions with polysaccharide directly translates to control of the ultimate size of formed nanogels [7]. A variety of techniques have been utilized to further control the ultimate size of nanogels, such as by changing the charge of the polysaccharide [8], by reducing the debye screening length with ions [9,10], or by reducing the tendency for forming disulfide bonds via chemical agents [11].

Associative interactions between proteins and polysaccharides are typically driven by electrostatic interactions among oppositely-charged, ionized groups on each component [12]. Proteins and most polysaccharides possess weak acid or weak base functionalities so that their relative ionization is dependent upon the pH of the solution [12]. Furthermore, proteins contain both weak acid and weak bases on their surface, so that the net-charge of the protein surface is either negative or positive if it is above or below its isoelectric pH, respectively. It has been shown that interactions between proteins and polycations will occur at pH values slightly below the protein’s isoelectric point, as there are sufficient anionic residues on the protein surface to allow limited interactions with the polycations despite the presence of cationic residues [13]. At a given pH, there will then be a driving force for associative interactions between a protein and a cationic polysaccharide based upon the number of anionic residues on the protein surface and the number of cationic charges on the polysaccharide, all of which is defined by the solution pH and the relative quantity of proteins and polysaccharides in the mixture [14]. Thus, it is important to define interactions based on the solution pH and quantities of protein and polysaccharide, the latter of which is typically represented by a protein-to-polysaccharide ratio (*r*).

Associative interactions between polyelectrolytes in solution can also be impacted by the presence of non-interactive chains present on one of the polyelectrolytes, which is utilized in polymer research to create novel colloidal assemblies from copolymers [15]. Specifically, copolymers may be created with homogeneous chains of either charged or uncharged monomers as end-to-end ‘blocks’ or as “grafts” that are attached to the side of the main chain. Such copolymers have a tendency to associate with oppositely-charged polyelectrolytes, yet the presence of non-interactive segments forces the assembly to take on specific structural arrangements, such as spheres or cylinders [16]. In a recent study, the assembly of spherical structures was demonstrated from an association between protein and a block copolymer comprised of an anionic polysaccharide and poly(ethylene glycol) (PEG) [17]. Such interactive complexes should also be formed if the polysaccharide component is cationic, although this has not yet been explored. Furthermore, no studies have determined the influence of a non-interactive chain on the ability of a charged polysaccharide to change the tendency of the associative protein to aggregate or to form nanogels.

This study was then built upon two notions: (i) size of nanogels formed by heat treatment of protein–polysaccharide complexes is impacted by the degree of electrostatic association between the polysaccharide and protein, and (ii) interaction between a charged polysaccharide and protein is impacted by the covalent attachment of PEG to the polysaccharide. It logically followed that the size of nanogels formed from protein–polysaccharide complexes would be indirectly controlled by covalently attaching PEG to the polysaccharide. Formation and physical properties of nanogels were determined using the model protein α-lactalbumin (α-lac) and the cationic polysaccharide chitosan (CH), a poly(glucosamine) previously used to create protein-based nanogels by thermal treatment [18]. It was hypothesized that interactions between α-lac and CH, ultimately defining the size of nanogels formed by thermal treatment, would be controlled by the covalent attachment of PEG to CH, the length of the CH chains (i.e., M_w(CH)_), and the relative amount of CH chains in relation to the amount of protein (*r*).

## 2. Results and Discussion

To determine the impact of the polysaccharide molecular weight and the presence of non-interactive polymer segments on the formation of nanogels, CH samples were prepared with average molecular weights of 113, 76, and 8.9 kDa with or without covalently attached 5 kDa polyethylene glycol chains (PEG), which would ultimately be utilized to prepare complexes with α-lactalbumin (α-lac) and form nanogels by thermal treatment. Complex formation between α-lac and CH has been attributed to electrostatic interactions between negative charges on α-lac and positive charges on CH [18]. Investigation of the ζ-potential of α-lac, CH, and covalently-linked graft copolymers of α-lac and CH (CHPEG) verified that all CH_XX_ and CH_XX_PEG samples were positively charged across the pH range of 3.5 to 7.5 while α-lac possessed a dominant positive charge between pH 3.5 and 4.5 and a dominant negative charge at pH 5 and above (Figure S1). There was no significant difference in the pH dependence of the electrical charge of CH_XX_PEG solutions when compared to CH_XX_ solutions, indicating that the relatively small degree of PEG substitution had little effect on the electrophoretic mobility of CH. Furthermore, no significant differences in ζ-potential values were noted between CH_XX_ samples or CH_XX_PEG samples with different molecular weights. Therefore, one would expect similar driving forces for electrostatic interaction and subsequent complex formation between α-lac and all of the CH_XX_ or CH_XX_PEG samples with increasing pH of mixed solutions.

Light scattering observations of mixed solutions at different *r*-values showed that significant interactions occurred between α-lac and CH_XX_ or CH_XX_PEG as the pH was adjusted from pH 3.5 to 4.3 (Figure S2). No significant differences were noted among the different samples, implying that the molecular weight, attachment of PEG chains, and even the *r*-value had no impact on the initial interactivity between positively-charged amine groups on chitosan and negatively-charged residues on the surface of α-lac. A previous study also found evidence of interactions between α-lac and CH above pH 4, validating these observations [19]. Based upon these findings, mixtures of α-lac and either CH_XX_ or CH_XX_PEG were prepared at different *r*-values and adjusted to pH 4.3, 4.8, 5.3, and 5.8, with the logic that the first pH represented a pH value where weak complexes formed and the other three pH values represented incremental increases of 0.5 pH units. These mixtures were subsequently heated at 70 °C for 20 min in a water bath to observe the potential formation of nanogels.

### 2.1. Turbidity of Heated α-lac/CH_XX_ or CH_XX_PEG Complexes

**Figure 1:**
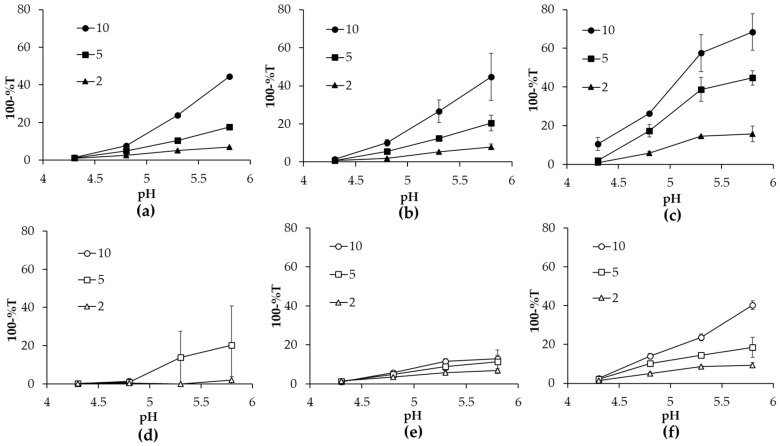
Effect of pH on turbidity of heated α-lac complexes at different *r*-values for mixtures with (**a**) CH_113_, (**b**) CH_76_, (**c**) CH_8.9_, (**d**) CH_113_PEG, (**e**) CH_76_PEG, and (**f**) CH_8.9_PEG. Note that datapoints are not shown for α-lac/CH_113_PEG for *r* = 10 at pH 5.3 and 5.8 due to formation of precipitates.

Turbidity measurements are good means to examine the colloidal properties of protein aggregates and have been previously utilized to study nanogels formed by heat treatment of protein–polysaccharide complexes [7]. Accordingly, turbidity values of heated mixtures of α-lac/CH_XX_ or CH_XX_PEG samples were determined as a function of *r*-value at pH 4.3, 4.8, 5.3, and 5.8 (Figure 1). In general, turbidity of heated mixtures increased with increasing pH and also with the *r-*value. The turbidity values of heated α-lac/CH_XX_ complexes were significantly greater than those of α-lac/CH_XX_PEG complexes of the same molecular weight, indicating the formation of aggregates with larger size and/or increased quantity. Heated mixtures with the lowest molecular weight CH (CH_8.9_ and CH_8.9_PEG) were more turbid when compared to mixtures with larger molecular weight CH for both non-PEGylated and PEGylated samples. For example, the most turbid mixture after heating at pH 4.8 was formed among mixtures with CH_8.9_ and *r* = 10 (Figure 1 and Figure S3). Turbidity consistently decreased a small amount among all samples after two weeks of storage, which could be attributed to gradual separation of large nanogels, dust, or bubbles (Table S1).

Reduced turbidity among heated α-lac/CH_XX_PEG complexes indicated that there was relatively less aggregation between α-lac during thermal treatment with the incorporation of the PEG chain. Aggregation during heat treatment of protein–polysaccharide complexes has been paired with the relative interactivity and subsequent protective actions of the polysaccharide against aggregation [6]. It is unlikely that CH_XX_PEG reduced α-lac aggregation by interacting more strongly with α-lac, since covalent attachment of PEG typically increases the hydrophilicity of polymers and decreases interactivity with proteins [20]. Thus, the reduced aggregation of α-lac implied that CH_XX_PEG provided an increased steric barrier with the hydrophilic PEG chains acting as a deterrent to protein–protein interactions during thermal treatment.

Low turbidity of heated mixtures at pH 4.3 could be attributed to relatively less aggregation occurring in these conditions with little additional impact of the interactive CH_XX_ or CH_XX_PEG. At pH 4.3, α-lac, CH_XX_, and CH_XX_PEG all possessed a net-positive charge (Figure S1); so, while the complexes would have been relatively weak, aggregation of α-lac during thermal treatment would also be relatively diminished. As the pH was increased, turbidity of the heated mixtures also increased because α-lac had more propensity to aggregate in proximity to its isoelectric pH. Turbidity increased further with increasing pH so that samples heated at pH 5.8 were the most turbid for each type of complex. Samples of heated α-lac without added CH_XX_ or CH_XX_PEG formed large, precipitating aggregates at pH 4.8, 5.3, and 5.8. Thus, the increased turbidity with increasing pH could be attributed to either a greater tendency for α-lac aggregation or to the promotion of aggregation by interaction with CH_XX_ or CH_XX_PEG.

Above pH 4.8, increased turbidity of heated mixtures with larger *r*-value could be attributed to the increased relative quantity of α-lac that could be free to aggregate within the complexes. Samples with relatively more CH_XX_ or CH_XX_PEG (i.e., *r* = 2) would then be less prone to aggregation because the interactive CH_XX_ or CH_XX_PEG remains with the protein during heating and prevents the protein from fully aggregating, as has been hypothesized from previous observations of heated protein–polysaccharide complexes [6]. Greater turbidity noted among samples with the relatively short CH_8.9_ or CH_8.9_PEG could be attributed to relative inability of the shorter chain lengths to inhibit α-lac aggregation because they conferred less of an electrosteric barrier when residing on the protein surface (Figure 1c,f). Surprisingly, this trend was not observed among heated α-lac/CH_113_PEG mixtures, where samples containing relatively more protein were more relatively more turbid (*r* = 5) or formed precipitates (*r* = 10) at pH 5.3 and 5.8 (Figure 1d), which were the only sample mixtures where precipitation occurred. CH forms a rigid-rod conformation through inter-hydrophobic interactions and hydrogen bonding [21,22]. Therefore, the low molecular flexibility among the CH_113_ and CH_113_PEG samples would have limited their ability to interact effectively with single proteins because they would be less capable of coiling and conforming to the curvature of the α-lac surfaces [23]. This factor did not appear to significantly impact aggregation among samples with CH_113_. However, in the case of CH_113_PEG, the presence of covalently-attached PEG would have further restricted interactivity with α-lac so that the protein was highly unprotected and free to form large, precipitating aggregates at high pH.

### 2.2. The pH Dependence of ζ-Potential of Heated α-Lactalbumin/CH_XX_ or CH_XX_PEG Complexes

**Figure 2:**
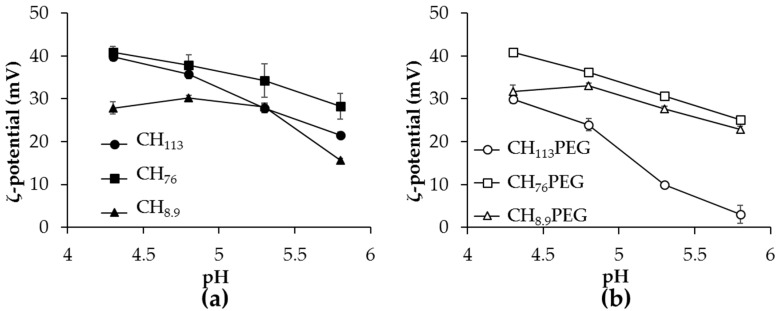
Effect of pH on the ζ-potential of heated α-lac mixtures (*r* = 10) with (**a**) CH_XX_ and (**b**) CH_XX_PEG.

In order to identify which set of heated mixtures would be more stable to further aggregation during storage, the electrical charge of heated mixtures with CH_XX_ or CH_XX_PEG was investigated as a function of pH and is shown for *r* = 10 in Figure 2. ζ-Potential of heated mixtures remained positive across the pH ranges studied, below and above pI of protein, indicating the remaining presence of positively-charged CH_XX_ or CH_XX_PEG on the aggregate surfaces. Magnitudes of ζ-potential were greater at relatively higher molecular weight of CH (CH_113_ and CH_76_). There were no noticeable changes to the measured ζ-potential of heated mixtures when comparing CH_XX_ (Figure 2a) and CH_XX_PEG samples (Figure 2b), except for samples with CH_113_PEG, indicating that the attached PEG chain did not impact the final charge signature among the aggregates.

The ζ-potential values of all mixtures studied, except the unstable CH_113_ PEG samples, were above +20 mV across the studied pH range (Figure 2). This high magnitude of surface charge is typically associated with an increased colloidal stability due to strong electrostatic repulsions between colloidal particles. The results were also in agreement with the turbidity observations, which showed that all samples, except CH_113_PEG, were stable to precipitation (Figure 1). There was a notable decrease in the magnitude of the positive charge among heated mixtures of α-lac/CH_113_PEG when the pH increased, which could be attributed to a small electrophoretic mobility because the samples were in the form of larger aggregates, as discussed in the preceding section.

### 2.3. Hydrodynamic Diameter and Morphology of Aggregates in Heated Mixtures

**Figure 3:**
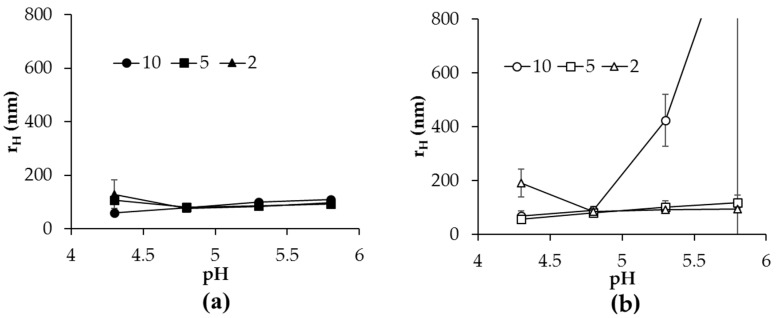
Effect of pH and *r*-value on hydrodynamic radii of heated α-lac mixtures with (**a**) CH_113_ or (**b**) CH_113_PEG.

Dynamic light scattering was utilized to investigate the hydrodynamic size of heated mixtures of α-lac and CH_XX_ or CH_XX_PEG to verify whether nanogel structures were created. Figure 3 demonstrates the effect of increasing *r*-value on average hydrodynamic radii of heated α-lac/CH_113_ or CH_113_PEG mixtures at pH values of 4.3, 4.8, 5.3, and 5.8. Average hydrodynamic radii of all samples increased gradually as pH increased and were insensitive to *r-*value above pH 4.3. For CH_113_PEG samples, average hydrodynamic radii became larger (>400 nm) with increasing pH, coinciding with a low-magnitude colloidal charge and the aforementioned formation of precipitates. All other tested mixtures were stable to precipitation and possessed average hydrodynamic radii on the order of 100 nm, suggesting the formation of nanometer-scale aggregates. Based upon prior studies with similar components and procedures [11,24,25], it was proposed that these aggregates are nanogels predominantly composed of α-lac with CH_XX_ or CH_XX_PEG at the surface and potentially also within the internal regions of the nanogels. Surprisingly, apart from the precipitates observed among CH_113_PEG samples at higher pH, there were no major differences in the observed hydrodynamic sizes of nanogels with changing molecular weight of CH (Figure S4).

Despite greater turbidity values among heated mixtures of higher pH, larger *r*-value, and in samples without covalently-attached PEG (Figure 1), there were no significant changes in the detected hydrodynamic size of microgels above pH 4.3 that did not form precipitates. Increased turbidity in the absence of chemical absorption could be attributed to either an increased size of colloidal objects, an increased refractive index, or an increase in the number of colloidal scatterers [26]. Thus, the lack of difference in detected hydrodynamic size suggested that all turbidity increases observed at increased pH, larger *r*-value, and among mixtures with CH_XX_ samples compared to CH_XX_PEG were due to a greater number of formed aggregates.

At pH 4.3, a few samples possessed a larger detected hydrodynamic diameter, but increased size was only consistently observed for CH_113_PEG mixtures with *r* = 2. It was not clear what caused the formation of nanogels with increased hydrodynamic sizes, although it could be potentially related to the lack of strong interactions between the components at pH 4.3. With weaker interactions, CH_XX_ or CH_XX_PEG coils would be only modestly tethered at the nanogel surface so that they would spread further out into the solution and occupy a greater total volume.

**Figure 4:**
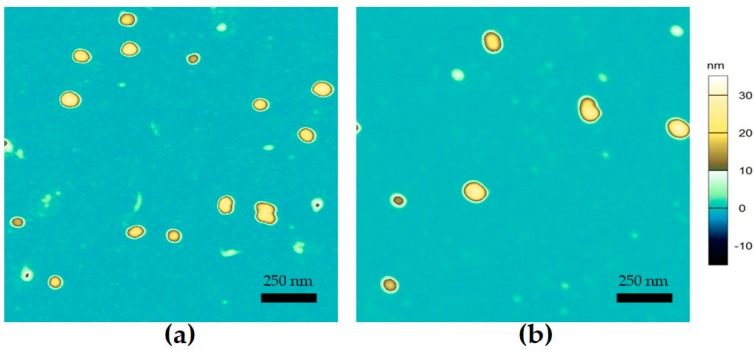
Images describing the topographical height of surface-deposited samples taken from heated α-lac mixtures (*r* = 2, pH 4.8) with (**a**) CH_113_ or (**b**) CH_113_PEG. Z-axis scale shown to right of (**b**).

In order to determine the morphology of nanogels formed from α-lac/CH_113_ or α-lac/CH_113_PEG (*r* = 2), mixtures heated at pH 4.8 were characterized by atomic force microscopy (AFM) (Figure 4). Morphology of the aggregates was determined by atomic force microscopy to be spherical and of a fairly homogeneous size distribution. Edge-to-edge size of the particles observed by AFM were on the order of 50–100 nm while heights of the particles were on the order of 20–35 nanometers, which are significantly smaller than detected hydrodynamic diameters of the same samples. The relatively small sizes observed by AFM imaging in air can be attributed to the significant shrinkage of microgels during drying for sample preparation, which has been previously observed [10]. Observed particles from α-lac/CH or α-lac/CHPEG heated mixtures were all spherical structures with no noted differences in morphology. There were no significant differences in either the size or morphology of any of the tested samples, which agreed with measurements of the hydrodynamic radii by light scattering.

## 3. Conclusions

Over all of the tested conditions, attached PEG led to no additional significant changes in the size or morphology of nanogels formed from the protein and CH, regardless of the molecular weight of the CH chain or the relative amount of added protein. This directly refuted the hypothesis, leading to the conclusion that addition of a non-interactive PEG chain to CH has no significant impact on the size of nanogels formed by thermal treatment of associative complexes between CH and α-lac. Reduced turbidity in mixtures heated with CH_XX_PEG indicated that PEG may have interfered with the interactions between protein during aggregation, reducing the total number of aggregates formed without significantly altering the typical size of nanogels. Turbidity results also indicated that PEG could interfere with interactivity and stability of α-lac with higher-molecular weight CH chains, prompting greater aggregation and precipitation. Apart from samples stabilized by the largest molecular weight CH_XX_PEG, nanogels formed with both CH_XX_ or CH_XX_PEG were consistently small (hydrodynamic radius ~100 nm) and possessed a strong positive-charge in low-acid conditions. Altogether, these findings implied that the addition of covalently-attached PEG chains to chitosan provided no benefits in terms of greater microgel production or specific size/shape inducement during thermal treatment of the constituent protein. Utility of the microgels as delivery vehicles or components of composite materials would be dependent upon their size but also their tendency to expand or contract in different solvation conditions, so further investigations are required to understand whether the covalently-attached PEG chain will confer any additional physical properties to the nanogels that could be of use in biomedical applications.

## 4. Materials and Methods

### 4.1. Materials

High purity CH (*M*_W_ = 113 kDa), monocarboxylated poly(ethylene glycol) (MPEG; *M*_W_ = 5 kDa), 1-ethyl-3-(3-dimethylaminopropyl)carbodiimide (EDC), and *N*-Hydroxysuccinimide (NHS) were purchased from Sigma-Aldrich (St. Louis, Saint Louis, MO, USA) and used as received. α-Lactalbumin (α-lac) was kindly donated by Davisco Food International from Le Sueur, MN. α-lac was further purified by dialysis (MWCO = 3500 Da) against ultrapure water (σ ≥ 18 MΩ·cm, Thermo Scientific, Waltham, MA, USA) for removal of minerals. Dialyzed α-lac was lyophilized and kept at −20 °C as dry power before use. Chemicals including acetic acid, sodium hydroxide, sodium nitrite, sodium chloride, sodium acetate, imidazole, hydrochloride acid, and deuterium chloride were purchased from Sigma-Aldrich (St. Louis, Saint Louis, MO, USA). Deuterium oxide were purchased from Cambridge Isotope Laboratories, INC. (Andover, MA, USA).

### 4.2. Different Molecular Weight of CH Preparation and Characterization

Portions of CH were depolymerized by oxidative degradation with 0.1 M sodium nitrite based upon the method of Mao et al. [27]. In brief, 1% (*w*/*w*) CH was prepared in 1% acetic acid aqueous solution and stirred overnight. Then, 27.79, 11.12, 4.45, 2.22, or 1.11 mL of 0.1 N sodium nitrite was added and allowed to react for 60 min at ambient temperature before neutralization with 1 N NaOH. The precipitated CH was collected by centrifugation, washed with deionized water, and finally lyophilized.

Characterization of the molecular weight (M_w_) of CH samples before and after oxidative degradation was performed by Size Exclusion Chromatography (SEC). The measurement was done on an Agilent 1260 high performance liquid chromatography system equipped with a Sephadex 200/30 combination column, an Agilent 1260 ISO pump, and an Agilent 1260 Refractive Index Detector (Agilent Co., Santa Clara, CA, USA). CH samples were dissolved at 1 mg/mL overnight in a pH 4.5 solution of 100 mM NaCl and 20 mM acetate and passed through a 0.45 µm pore size syringe filter prior to injection. Two hydrolyzed samples corresponding to 4.45 and 2.22 mL of added nitrite solution, as well as the non-hydrolyzed CH, were chosen for further testing, and their average molecular weights are reported in Table 1. From here onward, samples are referred to as CH**_XX_**, where XX refers to the determined molecular weight of each sample.

### 4.3. CH_XX_PEG Synthesis and Characterization

Covalent attachment of PEG chains to the depolymerized and untreated CH_XX_ samples was performed by a facilitated amidation reaction, as described by Huh et al. [28]. Briefly, CH_XX_ samples with different size were dissolved at a concentration of 10 mg/mL in 1% acetic acid solution and then diluted with the same volume of methanol. The coupling reactions between CH_XX_ and MPEG were achieved by using EDC and NHS as coupling agents at a CH_XX_:MPEG:EDC:NHS weight-based ratio of 500:250:48:25, which were allowed to react at ambient temperature for 24 h and terminated by dialysis against ultrapure water for three days to remove coupling agents and unreacted MPEG. PEGylated CH (CH_XX_PEG) samples in the retentate were lyophilized and stored dry at −20 °C until used.

The degree of deacetylation (DDA) of CH_XX_ and the degree of substitution (DS) of PEG on CH_XX_PEG were determined through 1D Proton Nuclear Magnetic Resonance (NMR) spectra by using a 300MHz NMR (Varian, Inova 300, Palo Alto, CA, USA) with a 5 mm quadrupole-nucleus probe. All samples were dissolved by using 2% wt DCl/D_2_O and then heated to 70 °C and cooled to room temperature right before the measurement. An example of the spectra for non-pegylated and pegylated samples of non-hydrolyzed CH_113_ is provided in Figure S5. Calculation of DDA was adopted from Hirai et al. [29] and Lavertu et al. [30], given as equation 1:

(1) $$DDA\;\left(\%\right)=\left[1-\left(\frac{1}{3}H_{\mathrm{ac}}÷\frac{1}{6}H_{2/6}\right)\right]\times 100$$

where H_2/6_ is the sum of integrated signals ascribed to protons at C-2 and C-6 positions (chemical shifted at 2.7 and 3.8 ppm) and H_ac_ is the integrated signal ascribed to the acetyl group (1.6 ppm) based upon the assignations of Jeong and others [31]. Quantification of DS was performed based on the method of Huh et al. [28] as the ratio of the integrated signals ascribed to methylene units of PEG (3.19 ppm) and CH saccharide residues (2.92 ppm) in 1D Proton NMR spectra. A summary of the DDA and DS for each CH_XX_ sample is provided in Table 1.

### 4.4. Solution Preparation

Buffer solution was prepared with 6 mM acetate and 3 mM imidazole at pH 3.5. Solution of 5 mg/mL α-lac was prepared by dissolving lyophilized α-lac in buffer for 4 h at ambient temperature. Solutions of 1 mg/mL CH_XX_ or CH_XX_PEG samples were prepared by dissolving lyophilized powders in buffer for 12 h at ambient temperature. Mixtures of α-lac and either CH_XX_ or CH_XX_PEG were prepared by mixing stock solutions with buffer at the desired proportion to achieve α-lac:CH_XX_ ratios *(r)* of 10, 5, or 2 with a constant combined α-lac and CH_XX_ content of 0.36 g/L and a final volume of 15 mL. This was achieved for mixtures with CH_XX_PEG samples by identifying the contribution of PEG to the average molecular weight via the determined DS values so that the amount of added weight of CH_XX_PEG (*W*_CHPEG_) to achieve a specified weight of CH (*W*_CH_) (i.e., a consistent molar ratio) was found by equation 2:

(2) WCHPEG=WCH(1+MW(PEG)∙(DS)MW(res))

where $M_{W\left(\mathrm{PEG}\right)}$ is the molecular weight of PEG and $M_{W\left(\mathrm{res}\right)}$ is the molecular weight of an average CH residue taking into account the quantity of de-acetylated glucosamine residues ($M_{W\left(\mathrm{res}\right)}=\left(DDA\right)\left(M_{W\left(\mathrm{glucosamine}\right)}\right)+\left(1-DDA\right)\left(M_{W\left(N-\mathrm{acetyl}\;\mathrm{glucosamine}\right)}\right)$. For each sample, the ratio *W*_CH_*:W*_CH-PEG_ was found to be approximately 1:3 since DS and DDA were similar among the prepared CH-PEG. Adjustment of the added weights in this fashion allowed direct comparison between mixtures with CH_XX_ and CH_XX_PEG, particularly since it was reasoned that only the CH component significantly contributes to interactivity with α-lac.

Nanogels were prepared from mixtures based on an established method [6]. Briefly, adjustment of the pH for all solutions and mixtures was performed using 1 and 0.1 N NaOH solutions. Aliquots were collected at desired pH values, placed in covered glass tubes, and stored at 4 °C until further analysis. Select samples were heated in a circulating water bath (Grant, Cambridge, UK) for 20 min at a temperature of 70 °C. After heating, samples were submerged in cold water and stored under refrigeration until their characterization.

### 4.5. Colloidal Sample Characterization

Colloidal surface charge of aqueous suspensions were determined by laser Doppler micro-electrophoresis using a Zetasizer Nano ZS light scattering instrument (Malvern, Worcestershire, UK) at a scattering angle of 173°. Average colloidal charge is reported as ζ-potential. Samples were measured at 25 °C in disposable plastic cuvettes. 

Turbidity of aqueous suspensions as a function of pH was also measured by a UV-Vis spectrophotometer (DU 730 Beckman Coulter, Fullerton, CA, USA) at a wavelength of 450 nm. The results of turbidity were presented as *100-T%*, where *T* is the light transmitted through the sample in the cuvette. Buffer of 6 mM acetate, 3 mM imidazole was used as reference blank.

Average hydrodynamic radii (*r*_H_) of heated mixtures were determined by dynamic light scattering with an ALV-CGS3 light-scattering goniometer (ALV, Langen, Germany) at a scattering angle of 90 degrees. *r*_H_ reported in the figures were calculated from the *z*-average diffusion coefficients using the Stokes–Einstein equation, where diffusion coefficients were determined using the CONTIN algorithm. All samples were diluted in buffer until concentration dependence was no longer observed in order to eliminate multiple scattering effects. 

Complex morphologies of selected samples were characterized with an MDP-3D atomic force microscope (Asylum Research, Santa Barbara, CA, USA) based upon an established method [11]. Samples were characterized in a dry state to reduce measurement noise and maximize surface attachment. Each sample was prepared by depositing 50 µL of diluted aqueous suspension onto freshly-cleaved mica, drying it under a stream of filtered air, and storing it overnight in a desiccator. Topographical images were obtained using a silicon nitride cantilever tip with aluminum reflex coating, a nominal spring constant of 5 N/m, and a resonance frequency of 150 kHz.

### 4.6. Statistical Treatment

The results were presented as the average of duplicate or triplicate. Statistical significance was conducted by means of the student’s *t*-test. Displayed error bars represent the standard deviation from independent sample measurements.

## Figures and Tables

**Table 1:** Molecular weight, degree of deacetylation, and degree of substitution of CH_XX_ or CH_XX_PEG samples used in this study.

Sample	*M*_W_ (kDa) ^1^	DDA (%) ^2^	Name Post-Modification	DS (%) ^3^
CH_113_	113	77.46 ± 0.59	CH_113_PEG	6.78 ± 0.94
CH_76_	76	83.37 ± 0.44	CH_76_PEG	8.14 ± 1.41
CH_8.9_	8.9	77.20 ± 2.54	CH_8.9_PEG	9.94 ± 1.58

^1^ Weight-averaged molecular weight determined by size-exclusion chromatography as described in Section 4.2. ^2^ Degree of deacetylation determined by 1D Proton NMR as described in Section 4.3. ^3^ Degree of substitution determined by 1D Proton NMR as described in Section 4.3.

## Supplementary Materials

The following are available online at http://www.mdpi.com/2310-2861/3/2/14/s1, Figure S1: ζ-potential of α-lactalbumin, CH, or CH-PEG as a function of solution pH; Figure S2: Intensity of scattered light at 90 degree scattering angle for mixtures of α-lactalbumin and (a) CH_76_PEG or (b) CH_76_ at different *r*-values as a function of solution pH; Figure S3. Effect of *r*-value on turbidity of α-lac complexes heated at pH 4.8 for mixtures with CH_113_, CH_76_, CH_8.9_, CH_113_PEG, CH_76_PEG, or CH_8.9_PEG; Figure S4: Effect of *r*-value on hydrodynamic radii of detected colloids within heated mixtures of α-lactalbumin and (a) CH or (b) CH-PEG of different molecular weight at pH 4.8; Figure S5: 1D Proton NMR spectra and assignations relevant to the chemical structures for (a) CH_113_ and (b) CH_113_PEG in 8% DCl/D2O (*v*/*v*) at concentrations of 5 mg/mL; Table S1: Turbidity of heated α-lac/CH_76_PEG mixtures at different pH and *r*-value 1 day after preparation and 14 days after preparation.
