# Supplementary material for: Impact of Chitosan Molecular Weight and Attached Non-Interactive Chains on the Formation of α-Lactalbumin Nanogel Particles
Source: Gels. 2017 Apr 26;3(2):14. doi: 10.3390/gels3020014 (PMC6318683; doi:10.3390/gels3020014)
Supplement: Supplementary file 1 [file gels-03-00014-s001.pdf]

# Impact of Chitosan Molecular Weight and Attached Non-Interactive Chains on the Formation of α-Lactalbumin Nanogel Particles

**Juan Du, Young-Hee Cho, Ryan Murphy, and Owen Griffith Jones**

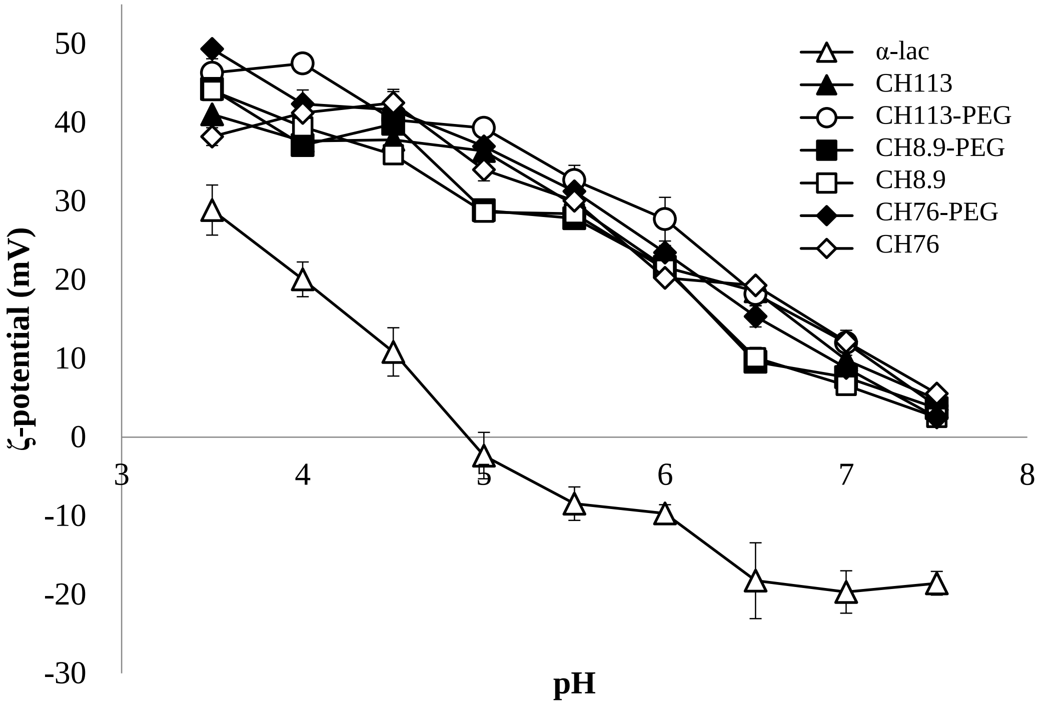


**Figure S1.** ζ-Potential of α-lactalbumin, chitosan (CH), or chitosan-*graft*-PEG (CH-PEG) as a function of solution pH.

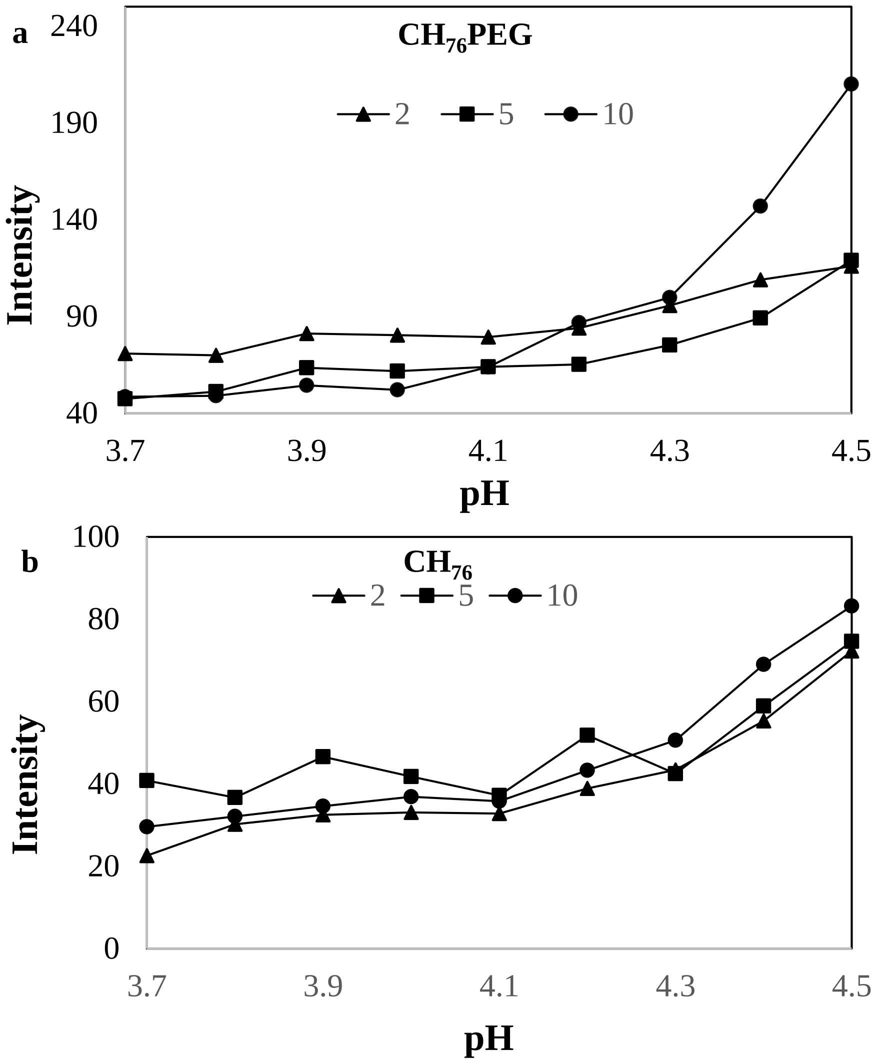


**Figure S2.** Intensity of scattered light at 90 degree scattering angle for mixtures of $\alpha$-lactalbumin and (**a**) $CH_{76}PEG$ or (**b**) $CH_{76}$ at different *r*-value as a function of solution pH.

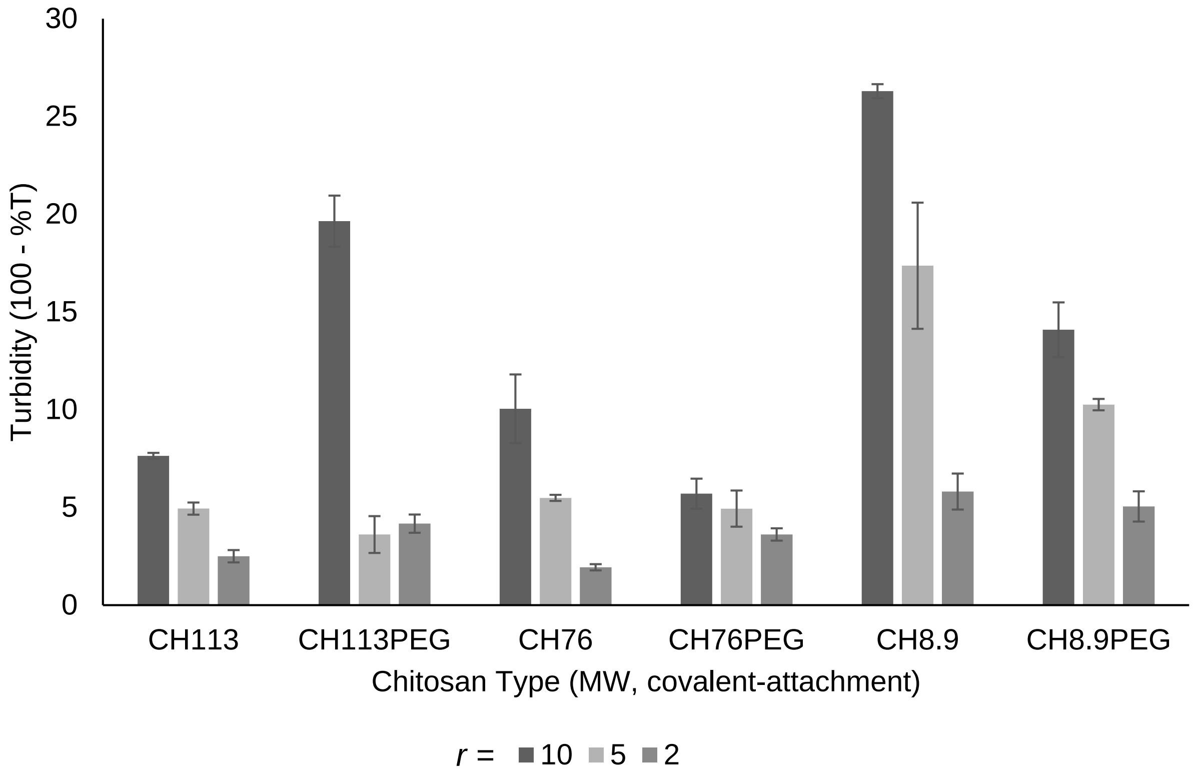


**Figure S3.** Effect of *r*-value on turbidity of $\alpha$-lac complexes heated at pH 4.8 for mixtures with $CH_{113}$, $CH_{76}$, $CH_{8.9}$, $CH_{113}PEG$, $CH_{76}PEG$, or $CH_{8.9}PEG$.

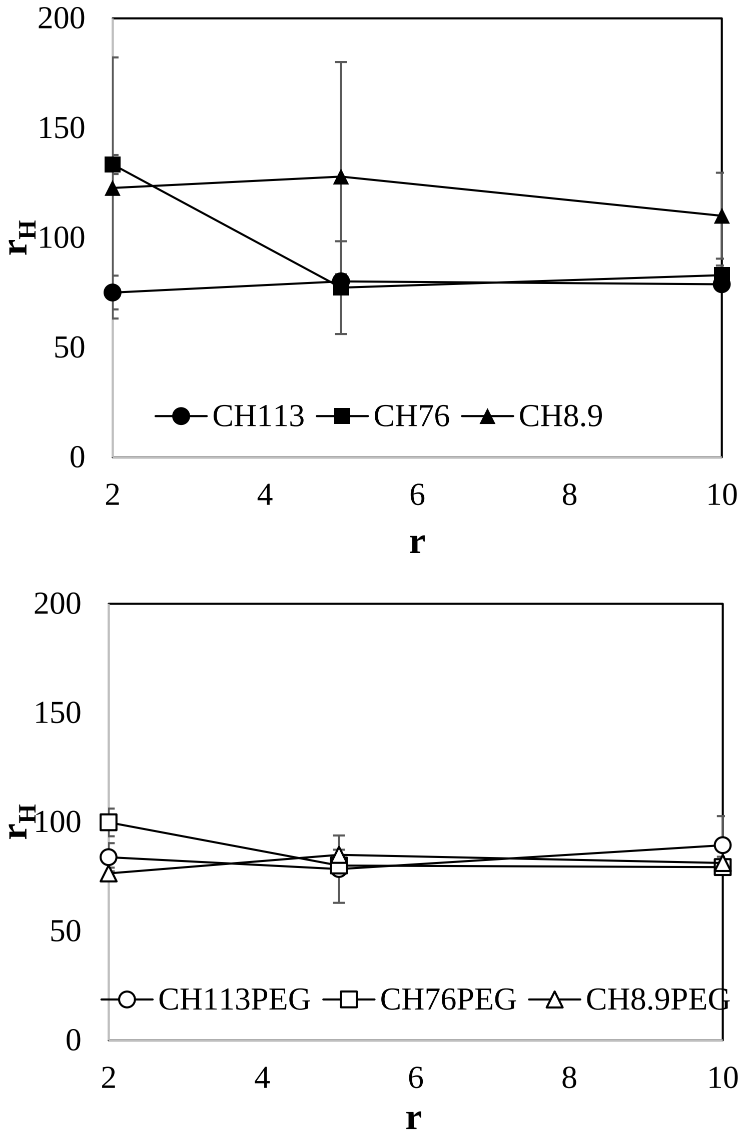


**Figure S4** Effect of *r*-value on hydrodynamic radii of detected colloids within heated mixtures of $\alpha$-lactalbumin and (**a**) CH or (**b**) CH-PEG of different molecular weight at pH 4.8.

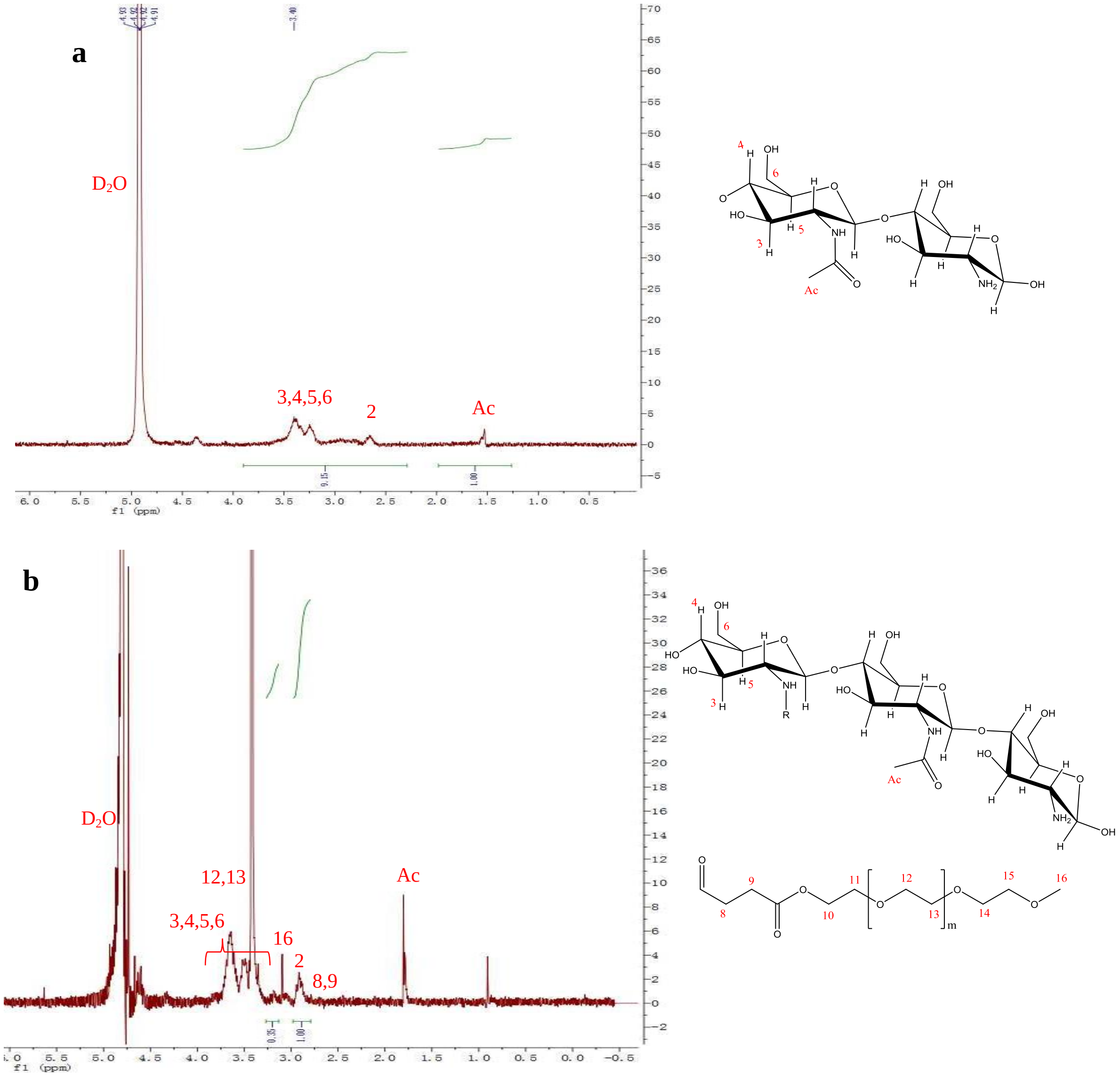


**Figure S5.** 1D Proton NMR spectra of (**a**) CH$_{113}$ and (**b**) CH$_{113}$PEG in 8% DCl/D$_2$O (*v*/*v*) at concentrations of 5 mg/mL. Schematics of the representative chemical structures and peak assignments are given on the right.

**Table S1.** Turbidity of heated $\alpha$-lac/$CH_{76}$PEG mixtures at different pH and *r*-value 1 day after preparation and 14 days after preparation.

| *r* | pH | Day 1 | Day 14 |
|---|---|---|---|
| | | 100-T% | 100-T% |
| **5** | 4.3 | 1.37 | 0 |
| | 4.8 | 5.59 | 2.95 |
| | 5.3 | 10.26 | 6.67 |
| | 5.8 | 15.67 | 10.26 |
| **2** | 4.3 | 1.60 | 0 |
| | 4.8 | 3.39 | 1.37 |
| | 5.3 | 6.46 | 2.50 |
| | 5.8 | 7.53 | 3.62 |

Table S1 shows that turbidity of heated mixtures decreased ~30%–60% after 2 weeks of storage (data at pH 4.3 was below noise threshold), possibly due to sedimentation of the largest nanogels in the suspensions and/or separation of dust or bubbles from sample tubes during storage. However, the general trend of turbidity between samples remained identical. This implied that mixtures with higher turbidity on day 1 possessed nanogels of larger average size and/or number compared to sample mixtures of lower turbidity, and this relation persisted following storage so that the same sample mixtures possessed nanogels of larger average size and/or number after 2 weeks.
